# Potato Juice, a Starch Industry Waste, as a Cost-Effective Medium for the Biosynthesis of Bacterial Cellulose

**DOI:** 10.3390/ijms221910807

**Published:** 2021-10-06

**Authors:** Daria Ciecholewska-Juśko, Michał Broda, Anna Żywicka, Daniel Styburski, Peter Sobolewski, Krzysztof Gorący, Paweł Migdał, Adam Junka, Karol Fijałkowski

**Affiliations:** 1 Department of Microbiology and Biotechnology, Faculty of Biotechnology and Animal Husbandry, West Pomeranian University of Technology, Szczecin, Piastów 45, 70-311 Szczecin, Poland; daria.ciecholewska@zut.edu.pl (D.C.-J.); michal.broda@zut.edu.pl (M.B.); anna.zywicka@zut.edu.pl (A.Ż.); 2 Pomeranian-Masurian Potato Breeding Company, 76-024 Strzekęcino, Poland; 3 Laboratory of Chromatography and Mass Spectroscopy, Faculty of Biotechnology and Animal Husbandry, West Pomeranian University of Technology, Szczecin, Klemensa Janickiego 29, 71-270 Szczecin, Poland; daniel.styburski@zut.edu.pl; 4 Department of Polymer and Biomaterials Science, Faculty of Chemical Technology and Engineering, West Pomeranian University of Technology, Szczecin, Piastów 45, 70-311 Szczecin, Poland; psobolewski@zut.edu.pl (P.S.); krzysztof.goracy@zut.edu.pl (K.G.); 5 Department of Environment, Hygiene and Animal Welfare, Faculty of Biology and Animal Science, Wroclaw University of Environmental and Life Sciences, Chełmońskiego 38C, 51-630 Wrocław, Poland; pawel.migdal@upwr.edu.pl; 6 Department of Pharmaceutical Microbiology and Parasitology, Faculty of Pharmacy, Medical University of Wroclaw, Borowska 211a, 50-534 Wrocław, Poland; adam.junka@umed.wroc.pl

**Keywords:** bacterial cellulose, green technology, potato tuber juice, starch industry, waste product, biomaterials

## Abstract

In this work, we verified the possibility of valorizing a major waste product of the potato starch industry, potato tuber juice (PJ). We obtained a cost-effective, ecological-friendly microbiological medium that yielded bacterial cellulose (BC) with properties equivalent to those from conventional commercial Hestrin–Schramm medium. The BC yield from the PJ medium (>4 g/L) was comparable, despite the lack of any pre-treatment. Likewise, the macro- and microstructure, physicochemical parameters, and chemical composition showed no significant differences between PJ and control BC. Importantly, the BC obtained from PJ was not cytotoxic against fibroblast cell line L929 in vitro and did not contain any hard-to-remove impurities. The PJ-BC soaked with antiseptic exerted a similar antimicrobial effect against *Staphylococcus aureus* and *Pseudomonas aeruginosa* as to BC obtained in the conventional medium and supplemented with antiseptic. These are very important aspects from an application standpoint, particularly in biomedicine. Therefore, we conclude that using PJ for BC biosynthesis is a path toward significant valorization of an environmentally problematic waste product of the starch industry, but also toward a significant drop in BC production costs, enabling wider application of this biopolymer in biomedicine.

## 1. Introduction

Bacterial cellulose (BC) is a versatile biopolymer, most effectively synthesized by non-pathogenic bacteria, *Komagataeibacter xylinus*. Similar to plant cellulose, BC is a linear polysaccharide consisting of β-1,4-glucan chains [1]. However, in contrast to plant-derived cellulose, BC is characterized by flexibility and high chemical purity. Moreover, it does not contain lignins, pectins, or hemicelluloses, the presence of which significantly prolongs the purification process of cellulose from plants [2,3,4]. Additionally, BC has high mechanical strength and water holding capacity owing to its dense fiber structure [5]. Importantly, BC can be considered biocompatible and non-toxic, as well as biodegradable, owing to the activity of cellulase-producing organisms. As a result, BC is safe with regard to industrial applications, as well as for the environment [1,2]. Thanks to the above-mentioned unique properties, BC is versatile, having numerous and diverse applications: in food, cosmetics, pharmaceutical/biomedical, paper, and textile industries [1,2,3,4,5,6,7].

In the laboratory setting, BC production is a well-established, straightforward process. Given the proper media and growth conditions, bacteria eagerly produce cellulose membranes. Further, the whole process can be mechanized and, at least partially, automated. However, BC production on an industrial scale needs a reduction in costs due to the relatively expensive medium—typically Hestrin–Schramm (HS)—required for its biosynthesis. From an economic point of view, the less expensive the medium is, the higher the potential profits will be. In this regard, the optimal solution would be the use of industrial waste, which is dispensable for the manufacturer. Furthermore, this strategy has additional benefits from an environmental perspective, and therefore, repurposing waste products represents added value. In this context, Kurosumi et al. examined the possibility of effective BC production (up to 6 g/L of dry BC weight) from various waste fruit juices from oranges, pineapples, apples, grapes, and Japanese pears [8]. However, to obtain a high yield of the BC, additional nitrogen sources (such as yeast extract) had to be introduced to the aforementioned juices. Otherwise, BC yields were more than 3 times lower. Lima et al. optimized the method of obtaining BC in static culture, using sisal juice (an agro-industrial residue) as a substrate to produce a culture medium supplemented with sugars and yeast extract [9]. Revin et al. showed that the use of wheat or whey decoction can enable up to 3 times higher BC yield after 3 days of biosynthesis under dynamic conditions, as compared with conventional media [10]. In turn, Kongruang proposed a way to reduce the cost of BC production by using coconut and pineapple juices (rich in proteins, carbohydrates, and microelements) [11]. However, again, these media had to be supplemented with yeast extract. For the same reasons, Li et al. investigated the possibility of using wastewater from the process of candied jujube production [12]. The results of their research have shown that such post-production water was inexpensive to obtain; however, it required acid pre-treatment in order to obtain sufficient glucose content. The 3 h hydrolysis at 80 °C resulted in a 58% higher glucose content in the raw material, which translated into a high BC yield. Other natural ingredients, reported by several research groups, included *hydrolysates of* corn stalks or wheat, rice husk pre-treated with enzymatic hydrolysis, fruit and vegetable peels [8,11,13,14,15,16]. Summarizing the above information, it can be concluded that the main issues with the application of natural or waste substrates are related to the mandatory pre-processing stage or insufficient nutritional content. Both of these issues may significantly increase the total cost and extend the BC production process.

With these aspects in mind, we turned our attention to the potato industry. Potatoes are vegetables with high nutritional value and their extensive cultivation in Eastern Europe, China, and India translates into high availability and low price throughout the year cycle [17]. At the same time, the potato agro-industry generates substantial waste. Recently, Abdelraof et al. described their efforts to utilize potato peel waste for obtaining substrates for BC biosynthetic media [18]. To our knowledge, this is the only report concerning the use of potatoes or potato waste to produce BC. However, the processing of potato peels in order to obtain sufficient sugar content also required the use of acidic hydrolysis, with additional reagents, and high energy consumption (the hydrolysis process was carried out at a temperature of 100 °C). As a result, the potential industrial applicability and cost-effectiveness of this approach are limited.

In our approach, we selected potato tuber juice (PJ), another significant potato agro-industrial waste product. The most prominent producer is the starch industry, which in the European Union produces over 10 million tons of starch and starch derivatives, and more than 5 million tons of proteins and fibers each year [19]. During the manufacture of a single ton of potato starch, approximately 3.5 tons of PJ waste is produced. Further, the proper management of PJ and other post-production waste is a significant and unresolved technological problem for this industry [20,21]. Given the growing challenges presented by climate change and other global environmental problems, the proper management of potato agro-industrial waste should be considered a key public policy target for governments and the private sector. Thus far, only the use of potato wastewater to irrigate agricultural fields has been explored; however, it creates problems with soil clogging, loss of water permeability, and water eutrophication [22].

Therefore, the goal of the current study was to investigate if PJ can be used as the medium for BC synthesis. We compared the yield, morphology, chemical composition, sorption properties, mechanical strength, and cytotoxicity of BC obtained from PJ-based cultures with those in a dedicated HS medium. In this fashion, we demonstrate an environmentally friendly strategy toward valorizing a major agro-industrial waste product and the possibility of a significant drop in costs related to BC synthesis, which should correlate with the wider application of this biopolymer in a wide range of industrial branches, including biomedicine.

## 2. Results and Discussion

### 2.1. Characterization of PJ Medium

In the current study, we used potato tuber juice (PJ) as a culture medium for BC biosynthesis. Importantly, the PJ was obtained in the same way as during the industrial production of starch. Further, the PJ medium required no pre-treatment process or any additional supplementation with nutrient sources, with the exception of the addition of 1% (*v*/*v*) ethanol. Ethanol is typically supplemented into HS media because it is a stimulating/initiating factor for BC biosynthesis by *K. xylinus* [7].

The lack of necessity of supplementing PJ with additional nutrients arises from the high content of various, easily absorbed nutrients present in potato tubers. While starch is the main component of the dry mass of potatoes (approx. 2%), tubers also contain (1) approx. 2% of proteins (a source of amino acids for bacterial cells), (2) 0.3 to 0.6% of sugars (a carbon source), and (3) a wide range of vitamins (e.g., A, C, B1, B2, B6) and minerals (e.g., potassium, phosphorus, magnesium, calcium, sodium, iron, zinc, and copper) [23,24]. Further, numerous other natural substances can also be found in potato tubers, including lipids, organic acids, polyphenols, fiber, and glycoalkaloids [24]. All of these compounds are also present in potato tuber juice [20,25]. Owing to its high nutritional value, potato tuber juice has already been considered a potential substrate for the *Lactobacillus casei* biomass production, as a food additive, or as an ingredient for functional food production [26,27]. The biological activity of PJ can also be of value; it can be used in the treatment of bowel diseases, reducing inflammatory symptoms [24].

In commercial media dedicated to BC production, such as HS, usually one specific sugar, typically glucose, is supplied as the carbon source [28,29]. However, in the case of media prepared from waste substrates, more complex composition and the presence of different sugars should be expected [16]. Our analysis of sugar content in PJ medium diluted with water in a 1:1 ratio showed the presence of sucrose (4.0 g/L), glucose (3.5 g/L), fructose (2.4 g/L), and starch (<0.1 g/L). Thus, in comparison with the HS medium, which has 20 g/L of glucose, the concentration of glucose alone and the total sugar content in the PJ medium were clearly lower (3.5 g/L and 9.9 g/L, respectively). However, our analysis indicated that diluted PJ contained a 25% higher concentration of proteins than HS medium (1.62 g/L v. 1.33 g/L). Finally, the initial pH of the PJ medium (6.08) was comparable to that of the HS medium (6.19)—neither required any further adjustment of this parameter.

### 2.2. Quantification of K. xylinus Cells and BC Yield

**Figure 1:**
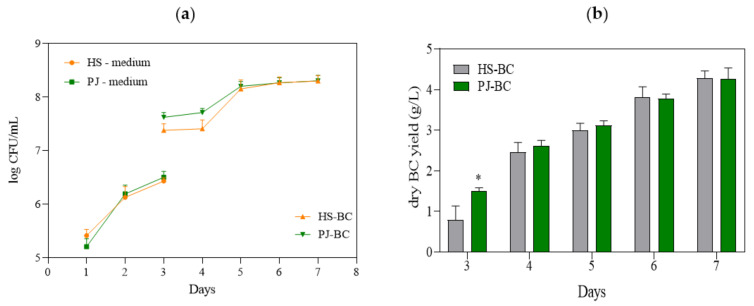
(**a**) Number of *K. xylinus* ATCC 53524cells in HS and PJ media and in BC pellicles; (**b**) yield of BC synthesized by *K. xylinus* ATCC 53524in HS and PJ media. Data are presented as a mean ± standard error of the mean (SEM). “*” indicates statistically significant difference between HS-BC and PJ-BC. HS—Hestrin–Schramm medium; PJ—potato juice medium; HS-BC—cellulose synthesized in HS; PJ-BC—cellulose synthesized in PJ.

The kinetics of bacterial growth and the dynamics of BC production constitute crucial factors for adjusting the optimal time and conditions of bacterial cultivation. As a result, both have a major impact on the economic aspect of the bioprocess and its scale-up [18]. In Figure 1a, the growth curves of *K. xylinus* in HS and PJ media are presented. As can be seen, the growth curves are comparable. Importantly, for both media, the *K. xylinus* began to grow without a distinct lag phase, indicating that this microorganism did not require a phase adjustment for replication. Molina-Ramirez et al. reported that the growth curve of cellulose-synthesizing bacteria correlates with an affinity for the carbon source [30]. Therefore, here, the affinity of *K. xylinus* American Type Culture Collection (ATCC) 53524strain for the carbon sources present in PJ medium was similar to glucose, the only carbon source in HS medium.

The most important result of the present study was the yield of BC, which—despite the substantial differences in the chemical composition of PJ and HS media—was comparable (4.3 g/L) (Figure 1b). It can be noted that on the 3rd day of cultivation in the PJ medium, the BC yield was significantly higher. This is likely due to a corresponding higher bacterial cell density (Figure 1a).

As already mentioned, we supplemented both our developed PJ medium and the control HS medium with 1% (*v*/*v*) ethanol. The effect of ethanol on BC yield was comparable regardless of the type of medium (Table 1). Further, we show that for the most efficient production of the BC, potato juice should be diluted with water (Table 1). This result is of particular importance considering the industrial starch production process, in which, depending on the technology used, potato juice can be diluted with water during starch separation or purification [20].

### 2.3. The Changes in pH and Chemical Composition of PJ during Fermentation Process

**Figure 2:**
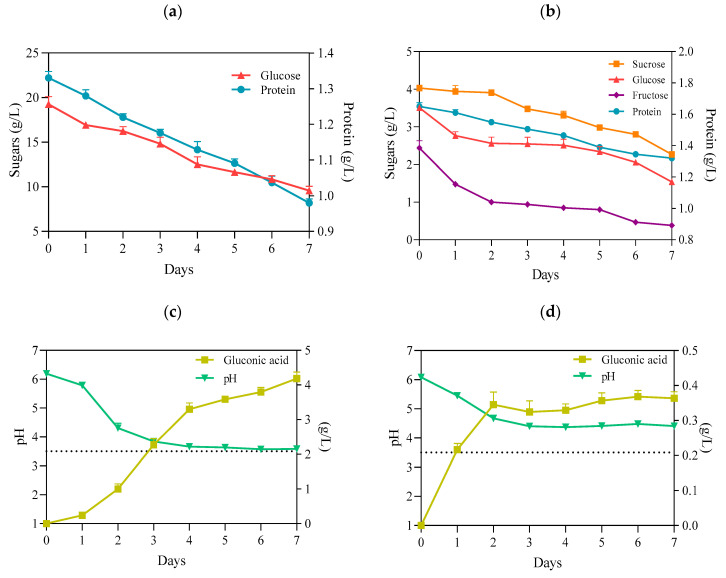
(**a**) Concentration of glucose and protein in HS medium; (**b**) concentration of sugars and protein in PJ medium; (**c**) pH and gluconic acid concentration in HS medium; (**d**) pH and gluconic acid concentration in PJ medium—all before and during BC biosynthesis by *K. xylinus* ATCC 53524. Data are presented as a mean ± standard error of the mean (SEM). critical pH value (3.5).

It is well established that *K. xylinus* can metabolize a variety of sugars, regardless of the presence of the preferred one [31]. Glucose is one of the main components of the HS medium and is the primary substrate for the synthesis of cellulose (Figure 2a). It is incorporated in a four-step metabolic pathway. First, in the cytosol, glucose is converted to UDP-glucose by uridine–diphosphate–glucose pyrophosphorylase (EC 2.7.7.64, UDP-glucose pyrophosphorylase). In the next step, cellulose synthase (EC 2.4.1.12), located in the bacterial cell wall, catalyzes the polymerization of UDP-glucose to poly β-1-4 glucan [32]. However, although the BC is an anhydrous polymer of glucose, *K. xylinus* can use other monosaccharides and disaccharides, such as fructose, xylitol, sucrose, maltose, or lactose [33]. This feature is also apparent in the present study (Figure 2b). Importantly, the use of glucose as the primary carbon source for *K. xylinus* is associated with a drawback. These bacteria can readily convert glucose to gluconic acid, significantly decreasing the pH (<3.5) of the medium (Figure 2c). This, in turn, results in a decrease in intracellular pH. As a result, a high concentration of gluconic acid can change the activity of metabolic pathways responsible for BC synthesis, reducing the yield [34,35]. In contrast, when the culture medium contains fructose, *K. xylinus* cells convert this monosaccharide to acetic acid, which results in a lower drop in pH, as compared with gluconic acid [36]. Therefore, the use of PJ, containing a mixture of sugars, including fructose, with a relatively low concentration of glucose (3.5 g/L), compared with the HS (20 g/L), resulted in a significantly reduced concentration of gluconic acid produced during fermentation, and the pH remained above 4, even at the end to the fermentation process (Figure 2d). Meanwhile, in cultures with HS medium, the concentration of gluconic acid at the end of the fermentation process was approx. 10 times higher, and the pH dropped to 3.5 (Figure 2c,d).

Nitrogen is a key component of proteins, making it necessary for cell metabolism. It comprises 8–14% of the dry cell mass of bacteria. Alongside carbon sources, proteinaceous nitrogen sources can also contribute to the enhancement of biomass production and BC synthesis, if suitably chosen [37]. The reports by several authors have shown that *K. xylinus* strains can utilize a wide range of protein and nitrogen sources, including casein hydrolysate, peptone, corn steep liquor, yeast extract, glutamate, soybean meal, glycine, and ammonium sulfate [7,37]. Likewise, in our present study, we observed that, although PJ and HS media differed in terms of qualitative and quantitative protein content, protein consumption during the BC biosynthesis process was similar (0.33 g/L v. 0.35 g/L) (Figure 2a,b).

The present study also confirmed that the starch was not metabolized during the BC biosynthesis process, as its concentration, 0.67 g/L, in media did not change during the cultivation of *K. xylinus*. Further, when using non-centrifuged PJ, containing starch solids, BC yield significantly decreased (Table 1).

### 2.4. The Role of Bacterial Strain and Presence of Sugars on BC Yield

**Figure 3:**
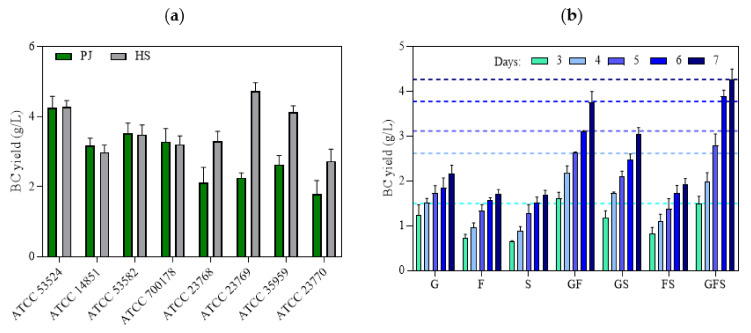
(**a**) BC yields of different *K. xylinus* strains in PJ and HS media after 7 days of cultivation; (**b**) BC yields of *K. xylinus* ATCC 53524 in HS medium with the addition of sugars at the same concentration as in PJ medium in the following days of cultivation. Data are presented as a mean ± standard error of the mean (SEM): G—glucose; F—fructose; S—sucrose. Horizontal lines in (**b**) indicate the BC yield of *K. xylinus* ATCC 53524 in PJ medium in the following days of cultivation.

The process of BC synthesis by bacterial cells is multilevel and includes many, tightly connected, metabolic pathways. A recent phylogenetic study of *Komagataeibacter* strains with defined genomes suggests that there is high variability in the structure of the pathways involved in BC synthesis [36]. The differences in structure and number of cellulose synthase operons in the genomes of *Komagataeibacter* strains are also considered the main reason behind inter-species differences in BC productivity and response to cultivation conditions [32,38]. Therefore, ideally, the culture medium used to produce BC should be adapted to the specific bacterial strain prior to the fermentation process [36,38]. On the other hand, considering the results reported by other authors related to the optimization of the culture medium, it can be noted that culture media with a more complex composition, particularly in terms of carbon and nitrogen sources, are more universal and result in relatively high yields of BC regardless of the bacterial strain used [7,30]. For this reason, we also aimed to determine whether the PJ medium can be considered universal for the cultivation of different *K. xylinus* strains. In four out of the eight tested strains (Figure 3a), BC yield using PJ medium was equivalent to HS medium. The variation in these results confirmed that BC production efficiency is strain dependent and closely related to the carbon source preference of the particular strain [31]. The role of intra-species variability in the effectiveness of synthesis of various biological-derived products, including BC, has been broadly discussed in the relevant literature [39,40,41,42]. Therefore, to enable replication and comparison with similar experiments by other research teams, we deliberately focused on the ATCC reference strains of *K. xylinus*.

Further, in order to determine which of the carbon sources present in the PJ plays a crucial role in the high BC yield, we prepared HS media supplemented with glucose, fructose, sucrose, or a combination of these sugars at the same concentrations as in PJ. The results showed that all of the sugars present in the PJ medium were metabolized and converted into BC by the *K. xylinus* ATCC 53524 strain (Figure 3b). The lowest BC yield was obtained when only a single sugar was present in the medium. Higher BC yields were observed for glucose–fructose and glucose–sucrose combinations. However, the highest BC yield (4.27 g/L) was obtained for the combination of glucose, fructose, and sucrose, which reflected the composition of the main sugars present in the PJ (Figure 2b v. Figure 3b). These results are in good agreement with previous studies that showed that the use of a combination of several different carbon sources in one medium resulted in substantially higher BC yields, as compared with the medium containing only a single one [43,44]. Further, among the reported carbon sources, glucose, fructose, and mannitol have been the best for BC production. One possible explanation for this is that cellulose-producing bacteria are able to convert structural glucose isomers (e.g., fructose) or the precursors of glucose (e.g., mannitol) into glucose [45]. An alternate sugar utilization strategy involves enzymatic cleavage of disaccharides to obtain glucose, which can explain why some bacterial strains have better BC production efficiency via sucrose or lactose utilization [31,37,45]. Clearly, there is no single pattern among cellulose-producing bacteria, and the selection of the most appropriate carbon sources for a given strain is crucial for efficient BC production [45,46].

### 2.5. Physicochemical Properties of BC Obtained from Potato Tuber Juice Medium

#### 2.5.1. Macro- and Microstructure

**Figure 4:**
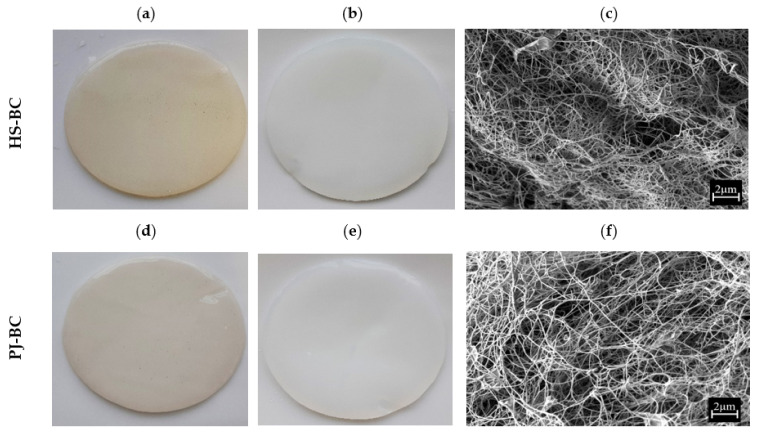
Macromorphology of BC from *K. xylinus* ATCC 53524: (**a**,**d**) before purification; (**b**,**e**) after purification; (**c**,**f**) micromorphology of BC from *K. xylinus* ATCC 53524 after purification, magnification 10,000× (SEM, Auriga 60, Zeiss, Oberkochen, Germany).

Macromorphological assessment of PJ-BC indicated that it has a homogenous, smooth surface with no visible residues of the culture medium. In fact, the micromorphology was similar to the typical micromorphology of mature BC synthesized in the HS medium (Figure 4a,b,d,e). Likewise, the microstructure of PJ-BC was similar to that of HS-BC (Figure 4c,f) and was consistent with that described in the literature [47]. These observations are particularly important given that the PJ medium used is typically a waste product. A frequent issue encountered with the use of other agro-industrial wastes, such as fruit and vegetable peels or juices (besides the previously discussed issues of pre-processing and/or insufficient nutritional content), is the high content of artifacts, solids, and/or pigments present in the raw substrates. These will then also be present in the medium and then also in the BC obtained.

**Figure 5:**
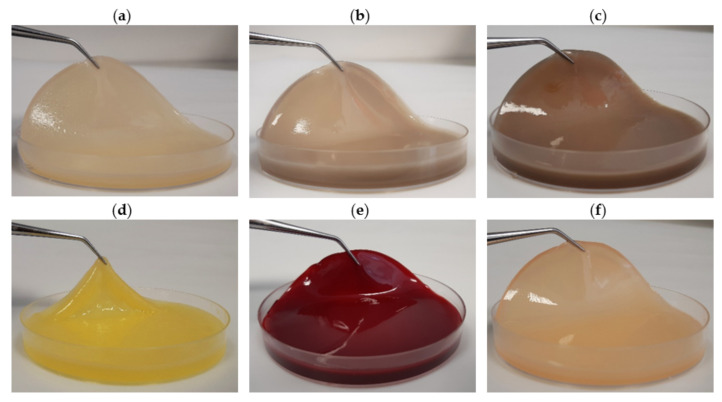
BC obtained from *K. xylinus* ATCC 53524 using (**a**) HS and natural ingredients-based media prepared from (**b**) potato juice, (**c**) potato peels, (**d**) orange peels, (**e**) beetroots, and (**f**) apples.

**Figure 6:**
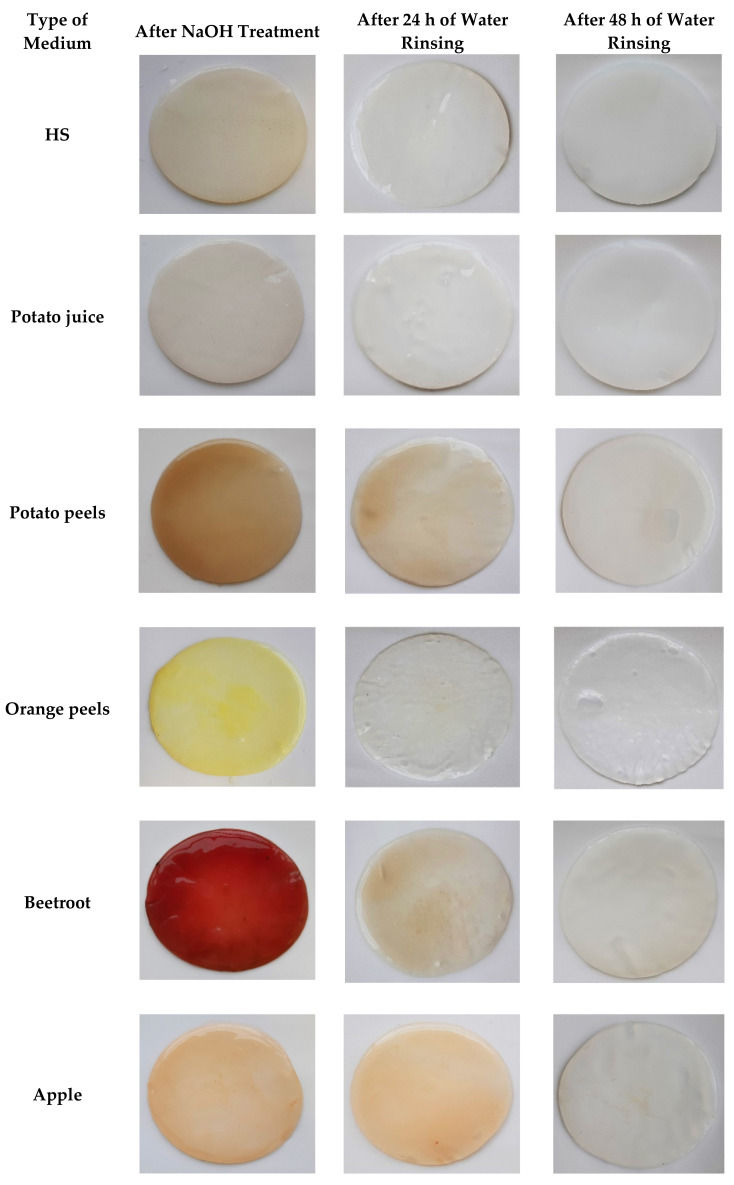
BC obtained from *K. xylinus* ATCC ATCC 53524 in HS and natural ingredients-based media in subsequent stages of purification.

**Figure 7:**
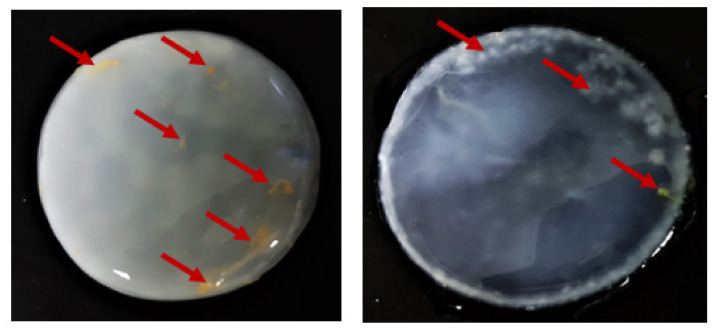
Purified BC from *K. xylinus* ATCC 53524 synthesized using natural (fruit) ingredients-based media. Red arrows indicate residues fragments (hard-to-remove impurities) of fruits bound in the BC membrane.

In order to assess the potential advantage of PJ over the other natural ingredient-based media, we prepared media from other food/agro-industry waste (potato peels, orange peels, beetroots, and apples) in the same fashion as the PJ media. We noted that, in terms of transparency, the PJ medium was comparable to the HS medium (Supplementary Figure S1). Further, for the case of PJ, there were fewer solids that had to be removed prior to the BC production. As was anticipated, the colors of the media prepared from the beetroots, apples, and even potato peels were also reflected in the pigmentation of the BC pellicles (Figure 5). This necessitated a longer purification process, as compared with the BC obtained from cultures in PJ or HS media (Figure 6). In the case of the BC obtained from an orange-peel-based medium, the yellow pigment was removed in the first stage of purification. However, in this case, the relative ease of pigment removal was likely due to the reduced thickness of the obtained cellulose membrane and its amorphous structure, which lowers its potential industrial applicability. In the case of other fruit-based media, some residues of the fruits were tightly bound to the BC and remained, even after the purification process (Figure 7). In contrast, none of these issues were observed when the PJ medium was used for BC synthesis.

#### 2.5.2. Analysis of ATR-FTIR Spectra

To further compare the BC obtained from PJ medium cultures to those in HS, we used infrared spectroscopy to examine chemical composition and crystallinity. The ATR-FTIR spectra of both HS-BC and PJ-BC exhibited all of the characteristic adsorption bands of cellulose functional groups (Supplementary Figure S2) that have been previously reported by other authors [48,49]. In all of the spectra, a pair of closely located bands at 710 cm^−1^ and 750 cm^−1^ was clearly visible and could be assigned to Iα and Iβ of BC microfibril allomorphs. All of the BC samples, regardless of the medium used, displayed a relatively high content of the Iα fraction (Table 2). Importantly, the use of the PJ medium did not influence the crystallinity indexes of the obtained BC, as compared with using HS. All of the samples exhibited high values of I.C. I1430/900. Likewise, the second crystallinity index, calculated as a ratio between the absorbance of the bands at 1370 cm^−1^ (CH bending) and 2900 cm^−1^ (CH and CH_2_ stretching) was also similar, regardless of the medium used.

#### 2.5.3. Water-Related Properties, Density, and Mechanical Properties of BC

For many applications, the water-related properties can be considered to be the most important features of BC, because they determine its absorption capacity and ability to retain and release liquid. Overall, our results (Table 2, Supplementary Figure S3) indicated that water-related parameters were comparable for both BC membranes, regardless of the medium used for their production. There were no statistically significant differences between the results of the total swelling ratio and water holding capacity. Likewise, regardless of culture medium, we observed no significant differences in density nor tensile mechanical properties (Table 2).

In summary, all of the obtained results were in good agreement with values reported in the literature for typical, unmodified BC [50]. In this context, it should be noted that the physicochemical properties of BC are influenced by many factors, such as the cultivation conditions and time, in addition to the composition of the culture medium [51,52]. Therefore, the lack of any negative impact of PJ medium ingredients on BC properties is of paramount value for its future applications. This is of particular importance in the case of biomedical applications, such as wound healing materials, in which especially both BC liquid capacity and mechanical properties play essential roles [51,53].

### 2.6. BC Cytotoxicity Screening

**Figure 8:**
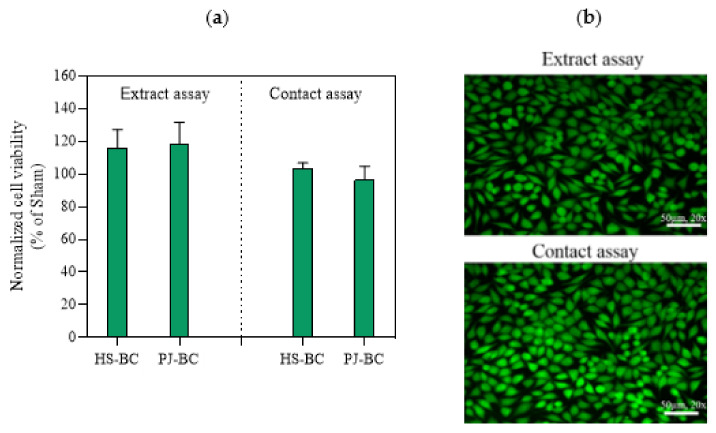
(**a**) L929 fibroblast viability data, as a percentage of the sham; (**b**) fluorescence live/dead visualization of L929 fibroblasts exposed PJ-BC for 24 h (extract and direct contact) and stained with Syto 9 (green, live) and propidium iodide dyes (red, dead). Data are presented as mean ± standard error of the mean (SEM); HS-BC—BC biosynthesized in HS medium; PJ-BC—BC biosynthesized in PJ medium.

BC has numerous potential biomedical applications including use as a biomaterial in tissue engineering, wound healing, and drug delivery [1,2]. In this context, it is important to note that BC is considered non-toxic. Therefore, it was important to confirm that BC obtained from PJ medium cultures was also not cytotoxic. We conducted extract and direct contact in vitro cytotoxicity assays, based on ISO 10993-5. In the extract tests, we observed robust growth of L929 cells exposed to all extracts. None of the tested materials resulted in viability below 70%, the threshold for cytotoxicity according to ISO 10993-5 (Figure 8a). There was no difference between PJ-BC and HS-BC. A similar trend was also observed in the direct contact assay. Importantly, the results of the viability assay were directly confirmed using fluorescence microscopy, with only live (stained green) cells observed (Figure 8b). Likewise, the morphology of L929 cells was not altered by either BC (Supplementary Figure S4). The results are in good agreement with our previous studies on BC-based materials [54]. However, we cannot compare our results with the BC obtained other by-product or waste media [10,11,16], because cytotoxicity studies are not (yet) a standard research practice in this context. We conclude that, from a cytotoxicity standpoint, the BC obtained from the PJ medium is equivalent to that obtained from conventional culture, making it suitable for biomedical applications.

### 2.7. The In Vitro Activity of PJ-BC and HS-BC Soaked with Antimicrobial against Two Species of Opportunistic Pathogens

**Figure 9:**
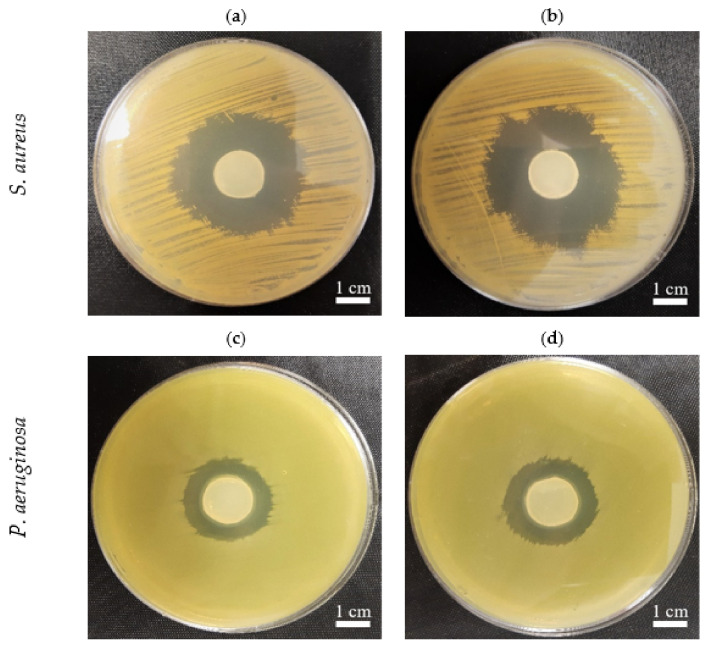
The growth inhibition zones in *S. aureus* (ATCC 6538) and *P. aeruginosa* (ATCC 15442) cultures, incubated with BC samples from *K. xylinus* ATCC 53524 soaked with an octenidine-containing antiseptic: (**a**,**c**) HS-BC; (**b**,**d**) PJ-BC. The comparable sizes of growth inhibition zones are seen with regard to specific pathogens independently from the type (PJ, HS-BC) of cellulose in which the sorption of octenidine was performed.

The use of BC for preparing primary wound dressings is one of the most thoroughly investigated and promising applications of this biopolymer in medicine. BC features flexibility, biocompatibility, and favorable water properties, thereby meeting the most crucial criteria and expert guidelines for modern dressings designed to treat chronic wounds. The relatively easy-to-perform introduction of locally active antiseptic into BC provides an additional promising avenue for chronic wound treatment. In fact, a number of studies have shown a high level of eradication of wound pathogens by antiseptic molecules released from BC, both in vitro and in vivo [54,55,56,57,58]. Further, an increasing number of such antiseptic-containing BC dressings are commercially available and applied in clinical practice [59]. Thus, we aimed to confirm that BC obtained from PJ media was also suitable in this context. We soaked PJ-BC and HS-BC with a modern antiseptic product (Octenisept^®^), containing octenidine dihydrochloride as the active ingredient, and assessed the antimicrobial activity against two species of opportunistic wound pathogens (*S. aureus* and *P. aeruginosa*) using a modification of the standard disk-diffusion assay. We observed no differences in the zones of growth inhibition, as a result of the octenidine released from PJ-BC or HS-BC (Figure 9). Thus, given the significantly lower cost of PJ-based medium, as compared with that of conventional HS medium, the overall advantage of the PJ-BC obtained here is clear. We conclude that PJ medium, particularly obtained as a waste from the potato starch industry, offers a cost-effective path toward broader and more common application of BC in biomedicine.

## 3. Materials and Methods

### 3.1. Preparation of Culture Medium

**Figure 10:**
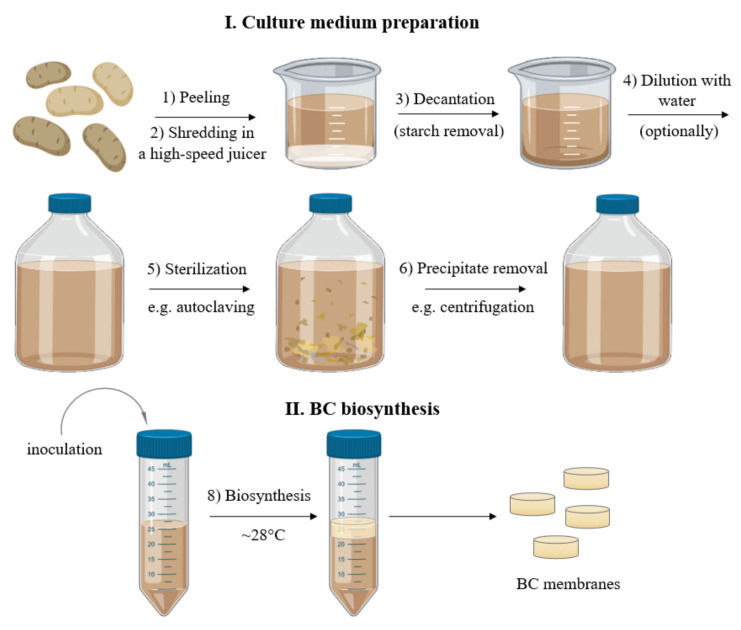
Scheme of the culture medium preparation and BC biosynthesis process.

For the current study, we selected the Tajfun variety potato (average dry matter content: 22%, average starch content: 16%), because it is one of the varieties used by the starch industry. Potato tubers were obtained from Pomeranian-Masurian Potato Breeding Company (Strzekęcino, Poland). Prior to use, the tubers were stored at 4 °C but for no longer than 4 weeks. Potatoes were washed, peeled, and then PJ was obtained using a high-speed juicer (Bosch, MES3500, Gerlingen, Germany). Following juicing, the PJ was left at room temperature for 60 min to enable the starch residues to settle, and then the potato juice was decanted. The decanted PJ was then diluted with distilled water in a 1:1 ratio, sterilized by autoclaving at 121 °C, centrifuged for 10 min at 1500× *g* to remove the remains of precipitated solids, and finally enriched with 1 *v*/*v*% of ethanol to yield the final PJ medium (Figure 10).

### 3.2. Determination of pH, Protein, and Carbohydrate Concentration in PJ Medium

The concentration of sugars, including sucrose, glucose, fructose in PJ medium was determined by liquid chromatography–tandem mass spectrometry (LC–MS/MS) technique (1260 Infinity II Series Liquid Chromatograph, Agilent, USA). An InfinityLab Poroshell 120 EC-C18 column (Agilent, Santa Clara, CA, USA), with a particle diameter of 2.7 µm, equipped with a guard column was used for the chromatographic separation. The mass spectrometer (Ultivo G6465B, Agilent, Santa Clara, CA, USA) coupled to the chromatograph was used to detect and identify the tested analytes. Quantitative analysis was performed based on calibration curves prepared with the use of high purity sugar standards (MilliporeSigma, Burlington, MA, USA).

The concentration of total protein was measured using the Bradford protein assay with bovine serum albumin as a standard [60]. The starch concentration was measured using the iodine starch method [61]. The measurements for both of these assays were performed spectrophotometrically at 595 nm and 615 nm, respectively, using an Infinite 200 PRO NanoQuant Microplate Reader (Tecan, Männedorf, Switzerland). The pH of the PJ medium was assessed using a laboratory pH meter (Elmetron, Zabrze, Poland).

### 3.3. Microorganisms and Culture Conditions

For BC production, a reference strain of *Komagataeibacter xylinus* (American Type Culture Collection, ATCC 53524) was used. A bacterial suspension of cell density equal to 2 × 10^5^ CFU/mL was used to inoculate 25 mL of medium in 50 mL plastic tubes (3.8 cm diameter, Polypropylene Conical Centrifuge Tube, Becton Dickinson and Company, Franklin Lakes, NJ, USA). Next, BC synthesis was conducted for 7 days at 28 °C. As a control, the standard HS medium (consisting of 2 *w/v*% glucose, 0.5 *w/v*% yeast extract, 0.5 *w/v*% peptone, 0.115 *w/v*% citric acid, 0.27 *w/v*% Na_2_HPO_4_, 0.05 *w/v*% MgSO_4_·7H_2_O), enriched with 1 *v/v*% ethanol, was used.

In order to fully assess the possibility of using the PJ medium and the full spectrum of its potential advantages, several additional experiments were performed in an analogous fashion. These included cultures of 7 additional *K. xylinus* strains from ATCC: 53582, 23770, 700178, 23768, 23769, 35959, 14851, as well as tests of several HS and PJ media combinations (PJ diluted with distilled water in 1:2 and 2:1 ratio; PJ and HS media without the addition of ethanol; non-centrifuged PJ containing starch solids; HS medium supplemented with the sucrose, glucose, fructose, and combination of these sugars at the same concentrations as in PJ). Additionally, we prepared and tested several media containing other ingredients that are agro-industrial wastes, including potato peels, orange peels, beetroot, and apples. The method of preparation of these media was analogous to the one applied for the preparation of the PJ medium. Finally, we also tested the use of other culture vessels such as Petri dishes 15 mm × 20 mm; the BC obtained from Petri dishes was also used for evaluation of its mechanical properties.

### 3.4. Determination of BC Yield

Starting from the 3rd day of cultivation, BC pellicles synthesized in HS (HS-BC) and in PJ media (PJ-BC) were harvested, purified by treating with 0.1 M NaOH solution at 80 °C for 90 min, and then rinsed with distilled water until pH became neutral (6.5–7.5). The obtained samples were then weighed on an analytical balance (accuracy 0.0001 g, WTB 2000 Radwag, Radom, Poland), dried at 60 °C, and weighed again. BC yield was expressed as dry mass (g) of BC/volume of culture medium (L).

### 3.5. Quantification and Viability Assessment of K. xylinus Cells

The quantity and viability of bacterial cells were determined in PJ and HS media (from 1st day of cultivation) and in PJ-BC and HS-BC pellicles (from 3rd day of cultivation) after their enzymatic digestion with cellulase, using alamarBlue Cell Viability assay (ThermoFisher, Waltham, MA, USA). AlamarBlue Cell Viability Reagent is a ready-to-use resazurin-based solution that functions as cell viability and metabolic activity indicator. For the digestion, the pellicles were washed in distilled water, transferred to 25 mL of the cellulase solution in citrate buffer (0.05 mol/L, pH 4.8), and incubated with shaking for 24 h at 30 °C. Next, the samples consisting of either culture medium or cell suspensions obtained from enzymatic hydrolysis of pellicles were centrifuged for 10 min at 3300× *g*. The resulting pellets were washed in phosphate-buffered saline (PBS, MilliporeSigma, Burlington, MA, USA), centrifuged again under the same conditions, and restored to their original volume with PBS. The bacterial suspensions (200 μL) were then transferred into wells of 96-well fluorescence microtiter plates (Becton Dickinson and Company, Franklin Lakes, NJ, USA), and 20 μL of alamarBlue reagent was added, followed by incubation for 1 h at 30 °C, in dark. The fluorescence was measured using a microplate fluorescence reader (Synergy HTX, Biotek, Winooski, VT, USA), using 540 nm excitation and 590 nm emission filters. Sterile PBS was used as the blank. As *K. xylinus* cells may show different metabolic activity depending on whether they are isolated from the medium or from the BC pellicle, dedicated standard curves were prepared separately for the cells obtained from the culture medium and the BC pellicles (Supplementary Figure S5). Using these standard curves, the fluorescence data were converted into log CFU/mL.

### 3.6. Assessment of pH, Protein, Sucrose, Glucose, Fructose, and Gluconic Acid Concentration during BC Biosynthesis

Each day of the fermentation process, samples of the culture media were taken and centrifuged for 1 h at 15,000× *g*. The supernatant was then separated from the pellet, filtered (PES 0.22 μm, MiliporeSigma, Burlington, MA, USA), and, if dedicated for LC-MS/MS analyses, additionally diluted with water (1:1000). All of the measurements were performed as previously described in Section 3.2 (Determination of pH, protein, and carbohydrate concentration in potato juice medium).

### 3.7. Evaluation of Physical and Chemical Properties of BC Obtained from PJ Medium

#### 3.7.1. Analysis of Microstructure Using Scanning Electron Microscopy (SEM)

Purified PJ-BC and HS-BC pellicles obtained after 7 days of culture were fixed in 1% (*v*/*v*) aqueous solution of glutaraldehyde for 0.5 h at room temperature and dehydrated using an ethanol dilution series from 10% to 100% (*v*/*v*) (5 min at each concentration). Subsequently, the samples were dried at room temperature for 15 min, coated with a 15 nm layer of carbon using a high vacuum carbon coater (ACE 600, Leica, Mannheim, Germany), and imaged with the ZEISS Auriga 60 scanning electron microscope (SEM, Auriga 60, Zeiss, Jena, Germany).

#### 3.7.2. Determination of Chemical Composition of BC Pellicles Using Attenuated Total Reflectance Fourier Transform Infrared (ATR-FTIR) Spectroscopy

Infrared spectra of purified PJ-BC and HS-BC pellicles obtained from 7 days cultures were evaluated using the ATR-FTIR technique, with an ALPHA FT-IR Spectrometer (Bruker Co., Leipzig, Germany) with a DTGS detector and the platinum–ATR-sampling module with a robust diamond crystal and variable angle incidence beam. For each BC pellicle, 32 scans at 4 cm^−1^ resolution were recorded over the spectral range of 4000–400 cm^−1^. Initial spectral data processing was performed using the Spectragryph 1.2 software package.

The crystallinity index was calculated using the ratio of absorbance values for peaks 1430/900 (Cr.R1) and 1370/2900 (Cr.R2). The fraction of the cellulose Iα was calculated from ATR-FTIR spectra according to Equation (1) [62]. The area of the peaks at A710 (710 cm^−1^) for Iβ and at A750 (750 cm^−1^) for Iα was determined from the spectra deconvoluted using Peakfit software. The following equation was used:

*f_α_* = A750/(A750 + A710) (1)

#### 3.7.3. Determination of Water Swelling Ratio

Purified PJ-BC and HS-BC pellicles obtained from 7 days cultures were dried at 60 °C for 6 h to remove any water, weighed, immersed in distilled water for 24 h, and weighed again. The swelling ratio as a percent of dry mass (SR%) was then calculated using Equation (2) as follows:

SR (%) = (W_w_ − W_d_)/W_d_ × 100 (2)

where W_w_ is the weight of the swollen BC and W_d_ is the dry weight of the sample.

#### 3.7.4. Determination of Water Holding Capacity

Purified and dried PJ-BC and HS-BC pellicles obtained from 7 days cultures were weighted, immersed in distilled water for 24 h to obtain maximum absorption level, and weighed again. The ability to hold water was determined using a moisture analyzer (Radwag, Radom, Poland) at 60 °C until the weight of BC was equal to the initial value (dry weight before hydration). Weight measurements were made automatically every 2 min. Water holding capacity (WHC) was then calculated using Equation (3) as follows:

WHC (%) = W_rw_/(W_w_ − W_d_) × 100 (3)

where W_rw_ is the weight of water retained in BC during drying, W_w_ is the initial weight of wet BC, and W_d_ is the dry weight of the sample.

#### 3.7.5. Determination of Density

The density of the dry, purified PJ-BC and HS-BC pellicles obtained from 7 days cultures was determined using a hydrostatic balance (XA 52/Y, Radwag, Radom, Poland) with methanol as a standard liquid. The weight of samples was measured at room temperature in the air as well as in methanol. The sample density was calculated using Equation (4) [63] as follows:

ρ = ρ_methanol_ × W_d_/(W_d_ − W_m_) (4)

where W_d_ is the weight of the dry sample in the air, and W_m_ is the weight of the sample in methanol.

#### 3.7.6. Evaluation of Mechanical Properties

The tensile tests were performed according to PN-EN ISO 527-1 using Instron Universal Testing Machine (Instron, Norwood, MA, USA). For this purpose, purified wet PJ-BC and HS-BC pellicles were obtained from 7 days cultures in Petri dishes, which were cut into 2.5 × 10 cm strips. Prior to testing, samples were gently pressed to remove excess water and obtain a uniform thickness of 4.5 to 5 mm. Tensile tests were then carried out at room temperature, with a crosshead speed of 10 mm/min. The average values of tensile strength and elongation at break were calculated from the stress–strain curves. All measurements were performed in four replicates.

### 3.8. Cytotoxicity Screening

In vitro cytotoxicity screening of the purified PJ-BC and HS-BC pellicles was performed using extract and direct contact assays, based on ISO 10993-5:2009 using ATCC CCL-1 (L929) murine fibroblasts (passages 10-28), as described previously [64]. L929 cell line, Dulbecco’s modified Eagle medium (DMEM), fetal bovine serum (FBS), L-glutamine, penicillin, streptomycin, and all other cell culture reagents were purchased from MilliporeSigma (MilliporeSigma, Burlington, MA, USA). All cell culture plasticware and disposables were purchased from VWR (VWR, USA). L929 cells were maintained and cultured in DMEM supplemented with 10% FBS, 2 mM L-glutamine, 100 U/mL penicillin. and 100 µg/mL streptomycin. For all cell culture assays, BC pellicles were sterilized by autoclaving.

#### 3.8.1. Extract Assay

For extract assay, 5 pellicles (~10 cm^2^) of each material were placed in a 6-well plate well, covered with 5 mL of growth media, and incubated for 24 h in a cell culture CO_2_ incubator at 37 °C. For dried HS-BC/PJ-BC films, 5 discs (~10 cm^2^) were covered with 3 mL of media. Finally, just media incubated in the same fashion served as a sham extract, while extracts of medical-grade PCL (CAPA 6430) and nitrile glove served as negative and positive (toxic) controls, respectively. In parallel, a 96-well plate was seeded with 1 × 10^4^ L929 cells per well and incubated for 24 h to allow for cell adhesion and spreading. At this point, the media was replaced with 100 µL of each extract, with 6 technical replicates performed per material. The plate was then returned to the incubator and cells were cultured for an additional 24 h, after which cells were examined using an inverted light microscope (Delta Optical IB-100). Cell viability was evaluated using resazurin assay [64]. Fluorescence measurements were performed using a fluorescent plate reader (Synergy HTX, Biotek, Winooski, VT, USA) at 540 nm excitation and 590 nm emission. Complete growth media in an empty well was used as a blank. The results were expressed as the percent of cell viability relative to control (sham) and were calculated using Equation (5) as follows:

% of cell viability = 100 × (FL_s_ − FL_b_)/(FL_c_ − FL_b_) (5)

where FL is the fluorescence intensity (arbitrary units) and indexes s, b, and c refer to sample, blank, and control, respectively.

#### 3.8.2. Direct Contact Assay

For the direct contact assay, 5 × 10^4^ L929 cells were seeded per well of a 24-well plate and incubated for 24 h to allow for cell adhesion and spreading. After this time, the media was replaced with fresh media, and BC pellicles (~2 cm^2^) that had been presoaked in cell culture media were placed directly on top of the cell monolayer (n = 5 pellicles per material). After a further 24 h of incubation, pellicles were carefully removed, and cell viability was evaluated as described above, using both light microscopy and the resazurin viability assay.

#### 3.8.3. Visualization of Fibroblast Viability

L929 fibroblasts cultured and treated as for the extract and direct cytotoxicity assays were stained for 15 min. with 3 µL of Syto 9 and Propidium iodide (PI) dyes (ThermoFisher Scientific, Waltham, MA, USA) 1000-fold diluted in PBS (MilliporeSigma, Burlington, MA, USA). Next, the dye-containing buffer was removed, and the cells were gently rinsed 3× times with PBS. Images of stained cells were captured using Lumascope 620 (Etaluma, Carlsband, CA, USA) at magnification ×20.

### 3.9. The In Vitro Activity of PJ-BC and HS-BC Soaked with Antimicrobial against Two Species of Opportunistic Pathogens

PJ-BC and HS-BC membranes were placed into wells of 24-well plates (F type, Nest Scientific Biotechnology, Wuxi, China) containing 1 mL of Octenisept^®^ (Schülke-Mayr, Norderstedt, Germany). The samples were incubated overnight at 4 °C. Cultures of reference *S. aureus* ATCC 6538 and *P. aeruginosa* ATCC 15442 (24 h in Tryptic Soya Broth medium (Biomaxima, Lublin, Poland)) were diluted in sterile 0.9% saline (Stanlab, Lublin, Poland) to 0.5 McF (DensiLaMeter II, Erba Lachema, Brno, Czech Republic) and spread evenly throughout Mueller–Hinton agar plates (Biomaxima, Lublin, Poland). The previously prepared PJ- and HS-BC soaked with Octenisept^®^ were then placed on top of the plates. The cultures were incubated overnight at 37 °C. The next day, the growth inhibition zones were recorded with the use of a digital camera.

### 3.10. Statistical Analyses

The data obtained in this study are presented as mean values ± standard error of the mean (SEM). Statistical differences between BC samples obtained in cultures using HS and PJ media were determined by one-way analysis of variance (ANOVA) and Tukey’s post hoc test. The cultures were conducted in triplicate and all experiments were repeated at least three times. Differences were considered significant at a level of *p* < 0.05. The statistical analyses were conducted using GraphPad Prism 9.0 (GraphPad Software Inc., San Diego, CA, USA).

## 4. Conclusions

Our results demonstrate that PJ (without any pre-treatment) is suitable as a source of nutrients for cellulose-producing *K. xylinus* bacteria. Most importantly, after diluting PJ with water 1:1 to prepare the medium, the yield of BC was equivalent to that obtained from a commercial HS medium. Further, the PJ-BC obtained in this study did not differ from conventionally produced HS-BC in terms of its physical and chemical properties and was not cytotoxic. Additionally, the release of the antimicrobial agent from both types of BC resulted in the similar growth inhibition of two opportunistic pathogens. As a result, PJ-BC should be able to be used in the same applications as commercially produced BC. Importantly, converting the BC production process to use PJ medium at an industrial scale should be relatively easy to implement, thanks to the high availability and low cost of PJ, a by-product of the potato starch industry.

## Figures and Tables

**Table 1:** Dry BC yield of *K. xylinus* ATCC 53524depending on the degree of potato tuber juice dilution with water, the addition of ethanol, and starch content.

	Dry BC Yield (g/L)
PJ:water dilution ratio 1:1 *	4.26 ± 0.32
PJ:water dilution ratio 1:2	1.97 ± 0.11
PJ:water dilution ratio 2:1	2.27 ± 0.18
HS with 1% of ethanol	4.28 ± 0.18
HS without ethanol	2.04 ± 0.37
PJ with 1% of ethanol *	4.26 ± 0.32
PJ without ethanol	1.97 ± 0.53
non-centrifuged PJ medium containing starch solids (with 0.67 g/L starch content)	1.689 ± 0.143
PJ medium prepared with decantation and centrifugation (with <0.1 g/L starch content) *	4.26 ± 0.32

Data are presented as a mean ± standard error of the mean (SEM). * are the same conditions, presented separately for purposes of comparison.

**Table 2:** Selected parameters of BC pellicles synthesized by *K. xylinus* ATCC 53524 in HS and PJ media.

	SR	WHC	EB	TS
HS-BC	299 ± 33	3.70 ± 1.24	20.0 ± 3.67	2.00 ± 0.26
PJ-BC	298 ± 33	4.07 ± 1.45	19.80 ± 3.28	2.05 ± 0.23
	*Iα* fraction	I.C.1_1370/2900_	I.C.2_1430/900_	ρ
HS-BC	0.44 ± 0.013	1.62 ± 0.21	1.04 ± 0.06	1.55 ± 0.02
PJ-BC	0.45 ± 0.018	1.57 ± 0.27	1.10 ± 0.11	1.49 ± 0.07

Data are presented as a mean ± standard error of the mean (SEM). SR—swelling ratio (%); WHC—water holding capacity after 8 min at 60 °C (%); EB—elongation at break (%); TS—tensile strength (MPa); ρ—density of BC (g/m^3^).

## Data Availability

The data that support the findings of this study are available from the corresponding author upon reasonable request.

## Supplementary Materials

The following are available online at https://www.mdpi.com/article/10.3390/ijms221910807/s1.
