# Supplementary material for: Potato Juice, a Starch Industry Waste, as a Cost-Effective Medium for the Biosynthesis of Bacterial Cellulose
Source: Int J Mol Sci. 2021 Oct 6;22(19):10807. doi: 10.3390/ijms221910807 (PMC8509763; doi:10.3390/ijms221910807)
Supplement: Supplementary file 1 [file ijms-22-10807-s001.zip › ijms-1396112-supplementary.pdf]

Supplementary material

Potato juice, a starch industry waste, as a cost-effective medium for the biosynthesis of bacterial cellulose

Daria Ciecholewska-Juśko [1], Michał Broda [1,2], Anna Żywicka [1], Daniel Styburski [3], Peter Sobolewski [4], Krzysztof Gorący [4], Paweł Migdał [5], Adam Junka [6], and Karol Fijałkowski [1,*]

[1] Department of Microbiology and Biotechnology, Faculty of Biotechnology and Animal Husbandry,
West Pomeranian University of Technology, Szczecin, Piastów 45, 70-311 Szczecin, Poland; daria.ciecholewska@zut.edu.pl (D.C.-J.); michal.broda@zut.edu.pl (M.B.); anna.zywicka@zut.edu.pl (A.Ż.)

[2] Pomeranian-Masurian Potato Breeding Company, 76-024 Strzekęcino, Poland

[3] Laboratory of Chromatography and Mass Spectroscopy, Faculty of Biotechnology and Animal Husbandry, West Pomeranian University of Technology, Szczecin, Klemensa Janickiego 29, 71-270 Szczecin, Poland; daniel.styburski@zut.edu.pl (D.S.)

[4] Department of Polymer and Biomaterials Science, Faculty of Chemical Technology and Engineering,
West Pomeranian University of Technology, Szczecin, Piastów 45, 70-311 Szczecin, Poland;
psobolewski@zut.edu.pl (P.S.); krzysztof.goracy@zut.edu.pl (K.G.)

[5] Department of Environment, Hygiene and Animal Welfare, Faculty of Biology and Animal Science, Wroclaw University of Environmental and Life Sciences, Chełmońskiego 38C, 51-630 Wrocław, Poland;
pawel.migdal@upwr.edu.pl (P.M.)

[6] Department of Pharmaceutical Microbiology and Parasitology, Faculty of Pharmacy, Medical University of Wroclaw, Borowska 211a, 50-534 Wrocław, Poland;
adam.junka@umed.wroc.pl (A.J.)

* Correspondence: karol.fijalkowski@zut.edu.pl; Tel.: +48 91-449-6714

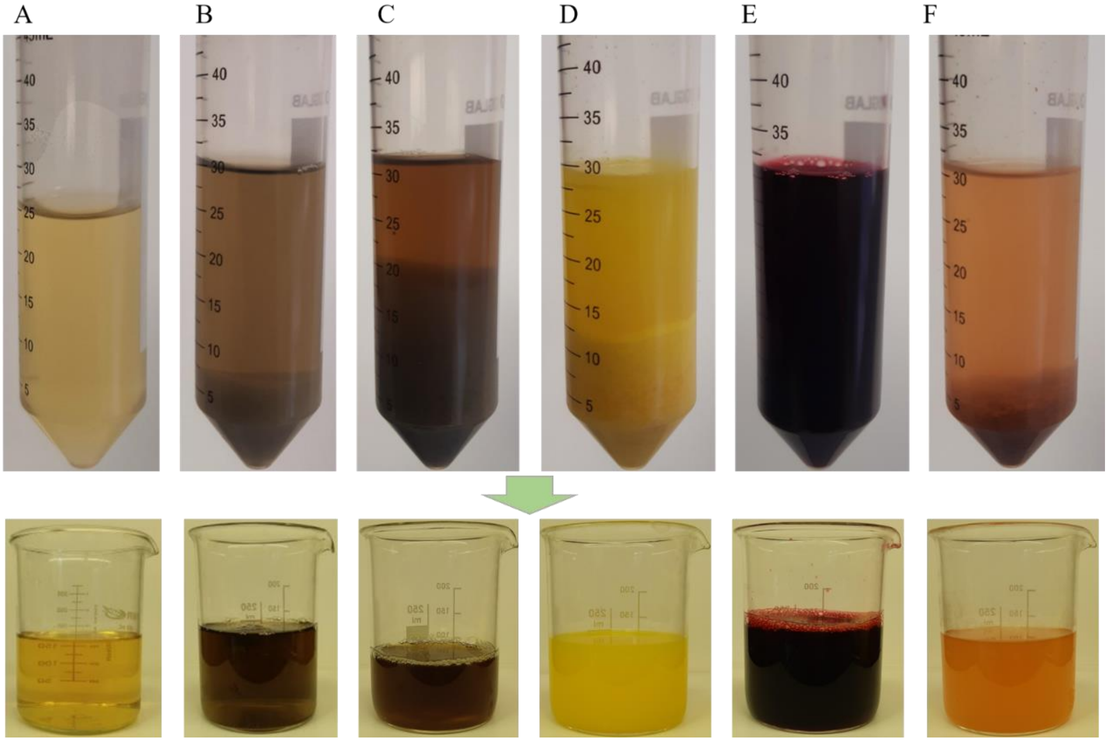

Figure S1. HS and natural ingredients-based media before (in 50 mL plastic tubes) and after decantation (in glass beakers); (**A**) HS; (**B**) potato juice; (**C**) potato peels; (**D**) orange peels; (**E**) beetroot; (**F**) apple.

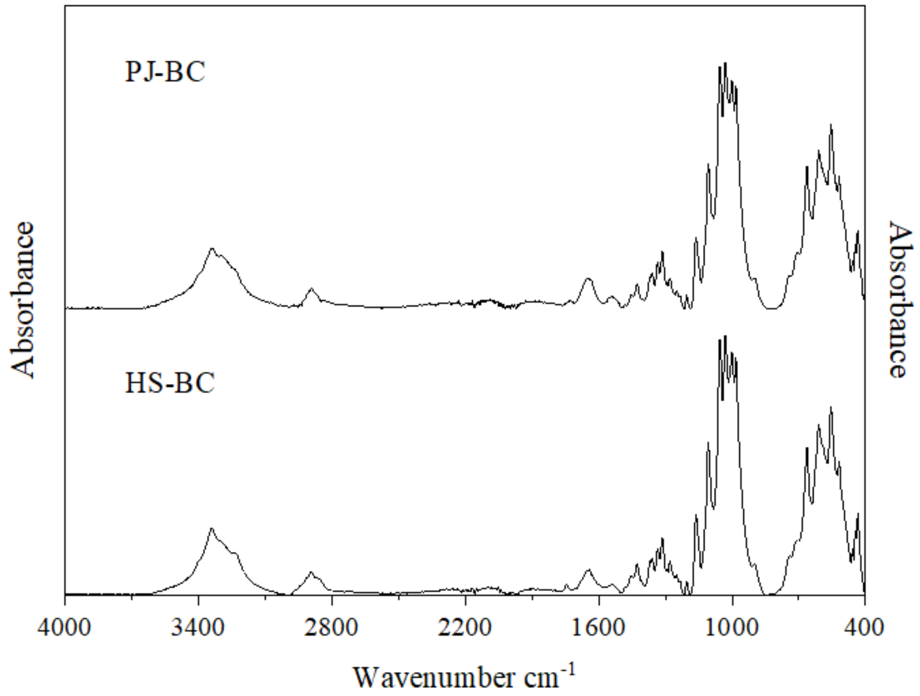

Figure S2. ATR-FTIR spectra of HS-BC and PJ-BC obtained from *K. xylinus* ATCC 53524 cultures.

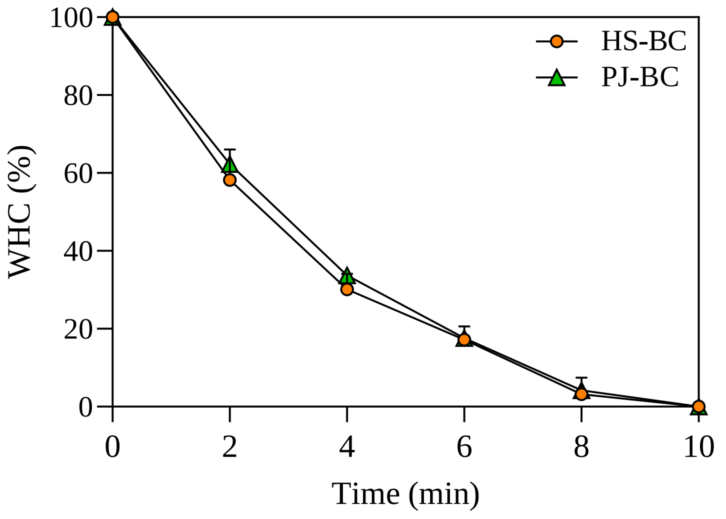

Figure S3. Water holding capacity (%) of HS-BC and PJ-BC obtained from *K. xylinus* ATCC 53524 during drying process at 60°C. Data are presented as a mean ± standard error of the mean (SEM).

Extract assay

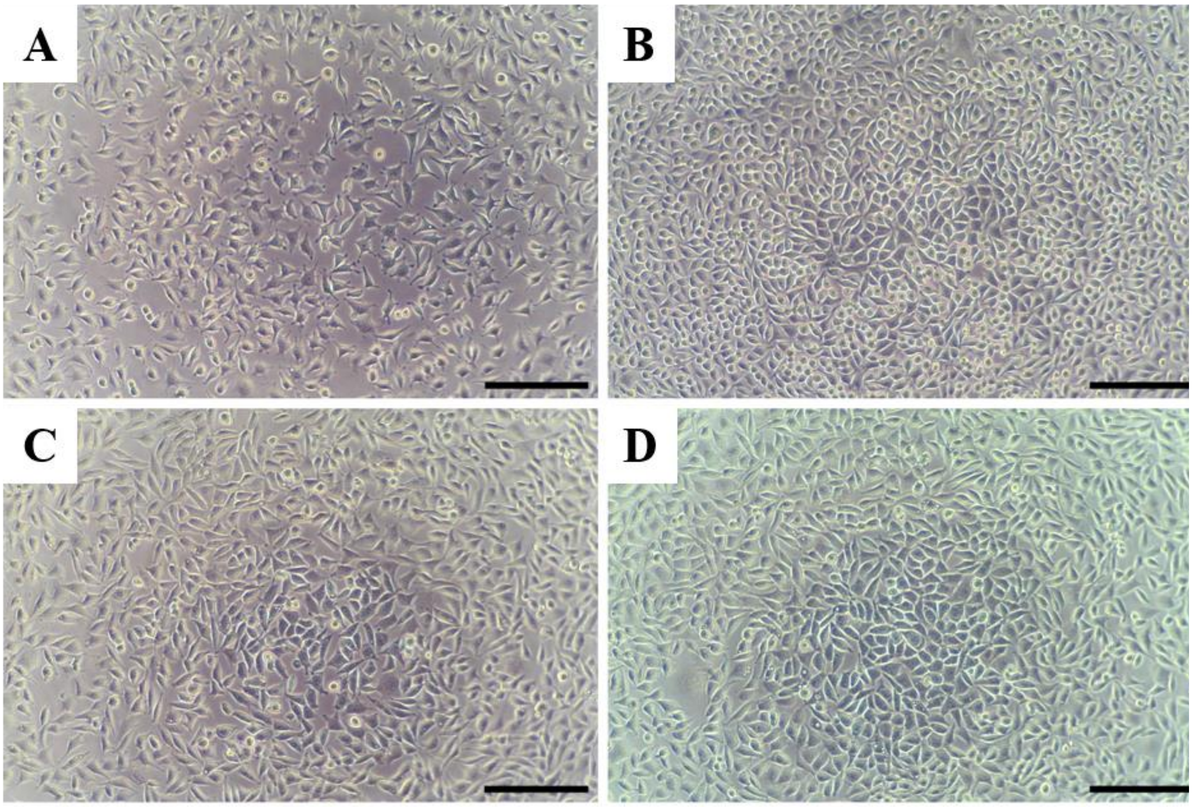

Contact assay

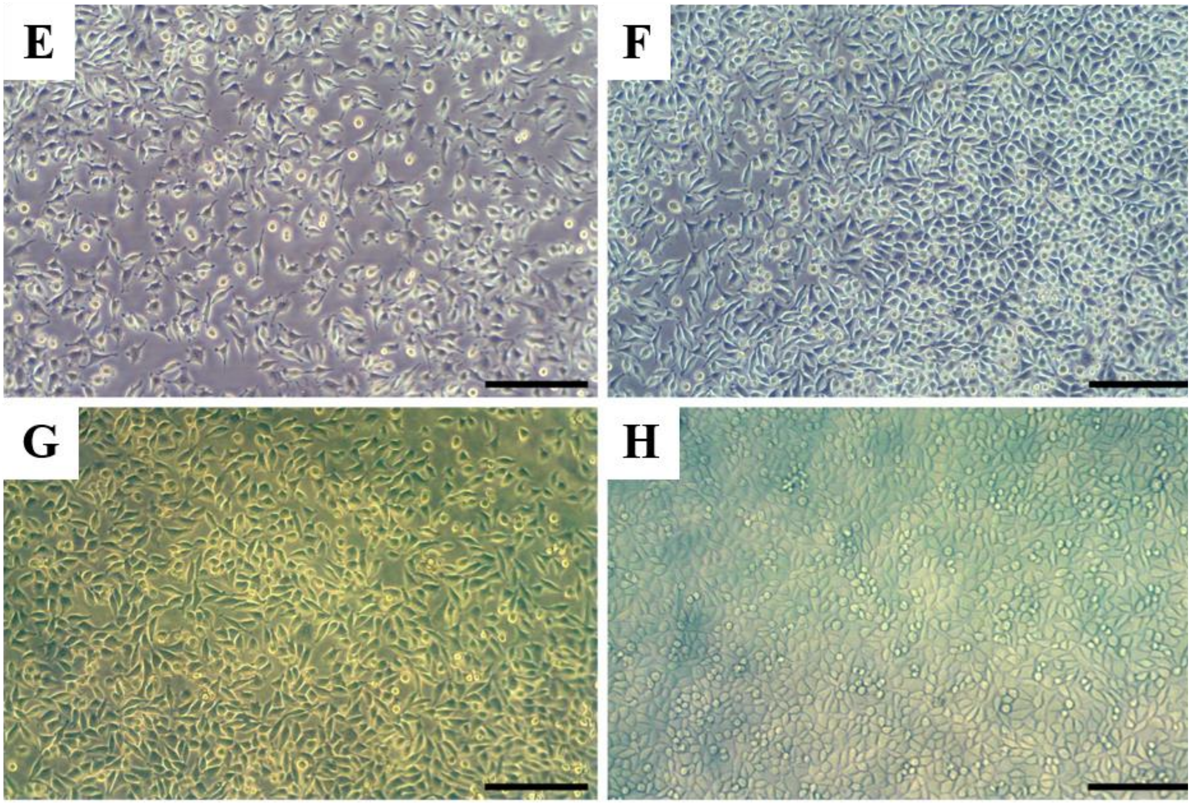

Figure S4. Representative micrographs of L929 cells. (**A**) 24 h after seeding (10000 cells per well); (**B**) After 24 h of incubation with sham extract; (**C**),(**D**) After 24 h incubation with extracts of HS-BC and PJ-BC obtained from *K. xylinus* ATCC 53524, respectively; (**E**) 24 h after seeding, prior to disc placement; (**F**) After incubation for 24 h without disc (sham); (**G**),(**H**) After 24 h incubation beneath discs of HS-BC and PJ-BC obtained from *K. xylinus* ATCC 53524, respectively. Images taken with discs in place - prior to removal. Scale bar indicates 200 μm.

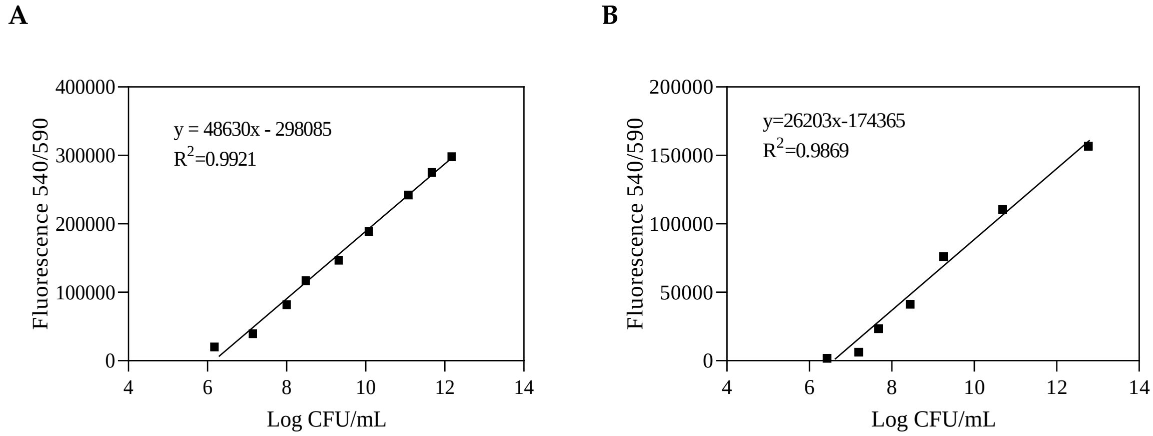

Figure S5. Standard curve of alamarBlue fluorescence intensity versus logarithm of *K. xylinus* ATCC 53524 cell concentration in the culture medium (**A**) and in the BC membranes (**B**).
